# Interface-Free Area-Scalable Self-Powered Electroluminescent System Driven by Triboelectric Generator

**DOI:** 10.1038/srep13658

**Published:** 2015-09-04

**Authors:** Xiao Yan Wei, Shuang Yang Kuang, Hua Yang Li, Caofeng Pan, Guang Zhu, Zhong Lin Wang

**Affiliations:** 1 Beijing Institute of Nanoenergy and Nanosystems, Chinese Academy of Sciences, Beijing, 100083, China; 2 School of Materials Science and Engineering, Georgia Institute of Technology, Atlanta, GA 30332, USA

## Abstract

Self-powered system that is interface-free is greatly desired for area-scalable application. Here we report a self-powered electroluminescent system that consists of a triboelectric generator (TEG) and a thin-film electroluminescent (TFEL) lamp. The TEG provides high-voltage alternating electric output, which fits in well with the needs of the TFEL lamp. Induced charges pumped onto the lamp by the TEG generate an electric field that is sufficient to excite luminescence without an electrical interface circuit. Through rational serial connection of multiple TFEL lamps, effective and area-scalable luminescence is realized. It is demonstrated that multiple types of TEGs are applicable to the self-powered system, indicating that the system can make use of diverse mechanical sources and thus has potentially broad applications in illumination, display, entertainment, indication, surveillance and many others.

Though today’s electronics have evolved to be more sophisticated than ever before, they still rely on power sources that maintain their operation. Self-powered technology provides a viable perpetual power solution by harvesting ambient energy of other forms. It has taken on a tremendous application prospect in portable, wearable and implantable devices as well as in stand-alone and remote electronics^1^,^2^,^3^,^4^,^5^. The overall efficiency of a self-powered system depends on not only the conversion efficiency of the energy harvester but also on how much energy can be actually extracted and then delivered to the load^6^. In this regard, an electrical interface must be added to regulate the electric output of the energy harvester so that the load matching can be achieved to optimize the useful output power^7^,^8^,^9^. For example, photovoltaics and thermoelectric generators normally require boosters that enhance their very low voltage to make them practically useful^10^,^11^,^12^. As another example, the recently developed triboelectric generator (TEG) that harvests mechanical energy features extremely high voltage but limited current^13^,^14^. When driving conventional electronics that need low voltage but high current, the TEG can only deliver a fraction of its optimum output power^15^, which is a major problem for practical applications of this technology. As a result, an electrical interface that includes voltage transformers is required to promote the current at the expense of the voltage^13^. However, it is highly desirable to remove the interface not only because the interface itself has a power consumption that further reduces the overall efficiency but also because the extra interface adds considerably additional size, weight and cost to the system.

Here, this work presents a self-powered electroluminescent system that is interface-free and area-scalable. The system takes full advantage of the high voltage of the TEG by directly connecting it to an alternating-current thin-film electroluminescent (ACTFEL) lamp. Induced charges pumped onto the lamp by the TEG can easily provide an electric field that is sufficient to excite luminescence because the ACTFEL lamp is voltage-driven instead of current-driven^16^. Since both of the two components own a capacitor-like structure that has infinitely large resistance, load matching is achieved without an electrical interface. Through rational serial connection of multiple ACTFEL lamps, effective and area-scalable luminescence is realized. It is demonstrated that multiple types of TEGs that can harvest energy from diverse mechanical sources are applicable to the self-powered system, indicating potentially broad applications of the self-powered system in illumination, display, indication, surveillance and many others.

## Results

**Figure 1:**
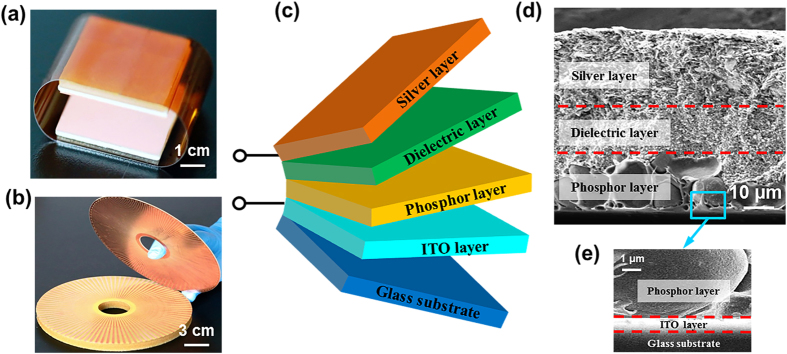
Architecture of the self-powered electroluminescent system. (**a**) Picture of the contact TEG. (**b**) Picture of the rotary TEG. (**c**) Schematic diagram of the ACTFEL lamp. (**d**) SEM picture of the cross-section of the lamp. (**e**) Enlarged view of the SEM picture that magnifies the ITO electrode layer.

The architecture of the self-powered system is illustrated in Fig. 1. It consists of two components, a TEG and an ACTFEL lamp. Here two types of TEGs are employed. They represent the two basic operating modes of the TEG^17^. The square-shaped TEG shown in Fig. 1a relies on the contact mode (Detailed fabrication process is provided in Methods), in which reciprocating pressing force perpendicular to the device results in pulsed electric output voltage and current. The detailed electricity-generating process was described in previous reports^18^. The disc-shaped TEG pictured in Fig. 1b has a stator-rotator structure, which belongs to the category of the sliding mode (Detailed fabrication process is provided in Methods). Continuous relative rotation between the stator and the rotator produces regularly alternating output current^13^. The stacked structure of the ACTFEL lamp is diagramed in Fig. 1c. On a glass substrate, four layers are fabricated in sequence, including an ITO electrode layer, a phosphor layer, a dielectric layer and a silver back electrode layer. The details of the structure are revealed in the SEM cross-sectional view (Fig. 1d,e). Complete fabrication process of the ACTFEL lamp is provided in Methods.

The nature of the electricity generation of the TEG is alternating flow of induced charges^17^. When two electrodes of a TEG and those of an ACTFEL lamp are connected respectively regardless of polarity, induced charges pumped by the TEG onto the ACTFEL lamp can form a fast-changing electric field within the phosphor layer, which accelerates electrons in the phosphor and excites luminescence^19^. On one hand, the TEG features alternating high voltage. On the other hand, the ACTFEL lamp is voltage-driven. Therefore, the output characteristics of the power source matches well with the need of the load, which enables an interface-free self-powered system.

**Figure 2:**
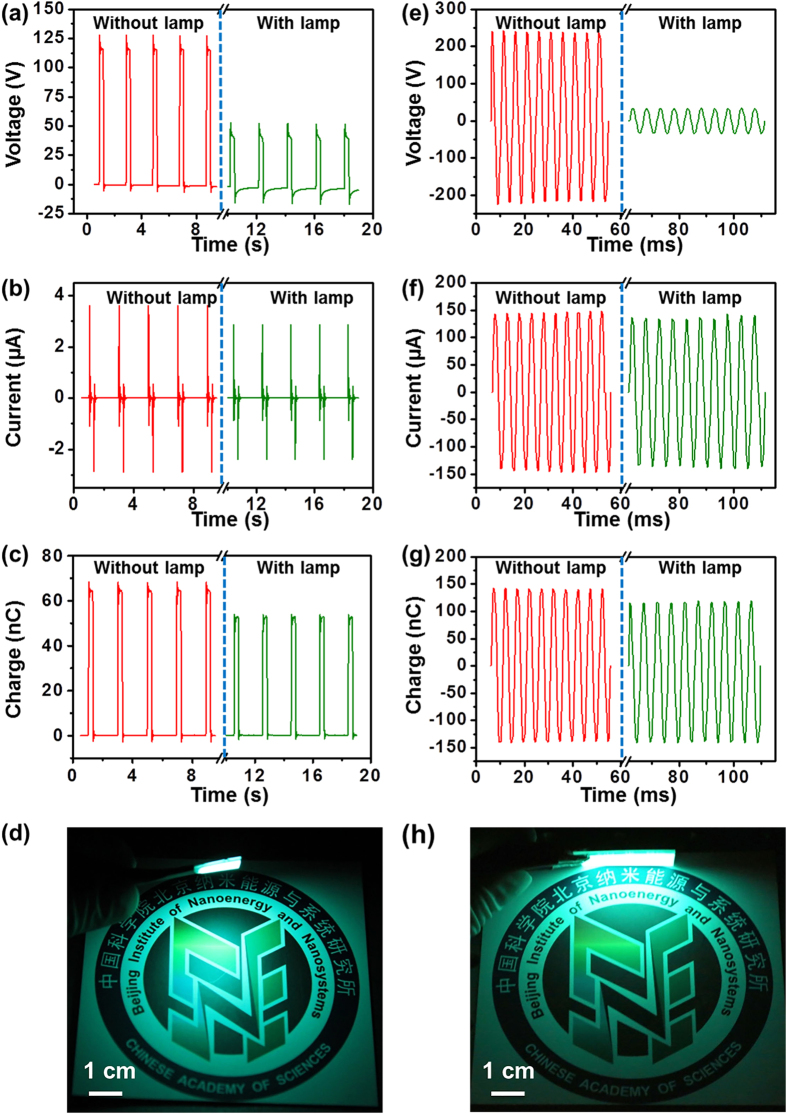
Electrical measurement results of the self-powered system when driven by two different kinds of TEGs. (**a**) Open-circuit voltage of the contact TEG (left column) and voltage applied onto the lamp (right column). Current (**b**) and induced charges (**c**) of the contact TEG in short-circuit condition (left columns) and those flowing through the lamp (right columns). (**d**) Picture of the electroluminescence by the contact TEG (permission is granted from the logo copyright holder). (**e**) Open-circuit voltage of the rotary TEG (left column) and voltage applied onto the lamp (right column). Current (**f**) and induced charges (**g**) of the rotary TEG in short-circuit condition (left columns) and those flowing through the lamp (right columns). (**h**) Picture of the electroluminescence by the rotary TEG (permission is granted from the logo copyright holder).

Before connecting to an ACTFEL lamp, a contact TEG (3 cm by 3 cm), when triggered by repeated reciprocating mechanical impact, could generate an open-circuit voltage of 125 V (left column in Fig. 2a). In short-circuit condition, the pulsed current has amplitude of 3.7 μA (left column in Fig. 2b), which carries induced charges of 65 nC for each peak as measured by an electrometer (left column in Fig. 2c). After an ACTFEL lamp (1.5 cm by 1.5 cm) was used as a load, the voltage actually applied onto the ACTFEL lamp has the same shape of square wave (right column in Fig. 2a) as that of the open-circuit voltage. The reason for the apparently reduced amplitude in Fig. 2a is because the voltage across the ACTFEL lamp is determined by the capacitance of the lamp, which is different from that of the TEG. The current (right column in Fig. 2b) and induced charges (right column in Fig. 2c) that flow through the ACTFEL lamp have slightly dropped amplitude compared to those in the short-circuit condition. This is because the capacitor-structured ACTFEL lamp poses a capacitive reactance and produces opposition to the current flow across the lamp. The induced charges pumped onto the ACTFEL lamp by the TEG exert a sufficiently high electric field that can excite transient luminescence of the blue-green phosphor^20^,^21^,^22^, as demonstrated in Fig. 2d.

The rotary TEG (10 cm in diameter) shown in Fig. 1b produces continuous alternating current as its two components have relative rotation^13^. The open-circuit voltage, short-circuit current and induced charges at a rotation rate of 500 r/min are displayed in the left columns in Fig. 2e–g, respectively. When an ACTFEL lamp (3 cm by 3 cm) is introduced to form a system, the voltage also significantly drops (right column in Fig. 2e). The constantly changing current from the TEG can pass through the load. It is noticed that the current amplitude (right column in Fig. 2f) and the induced charges (right column in Fig. 2g) are only slightly smaller than those in the short-circuit condition, which is in contrast to the case of the contact TEG. This deviation is originated from the different current frequency provided by the two types of TEGs. The time span of a current peak produced by the rotary TEG is derived to be 2.5 ms, while a single current peak resulting from the contact TEG is found to be 35 ms (Fig. S1). Therefore, the higher current frequency from the rotary TEG (250 Hz) leads to smaller capacitive reactance in the AC circuit and thus less reduced current amplitude. Driven by the rotary TEG, the ACTFEL lamp emits continuous luminescence, as shown in Fig. 2h and Supporting Movie S1.

## Discussion

**Figure 3:**
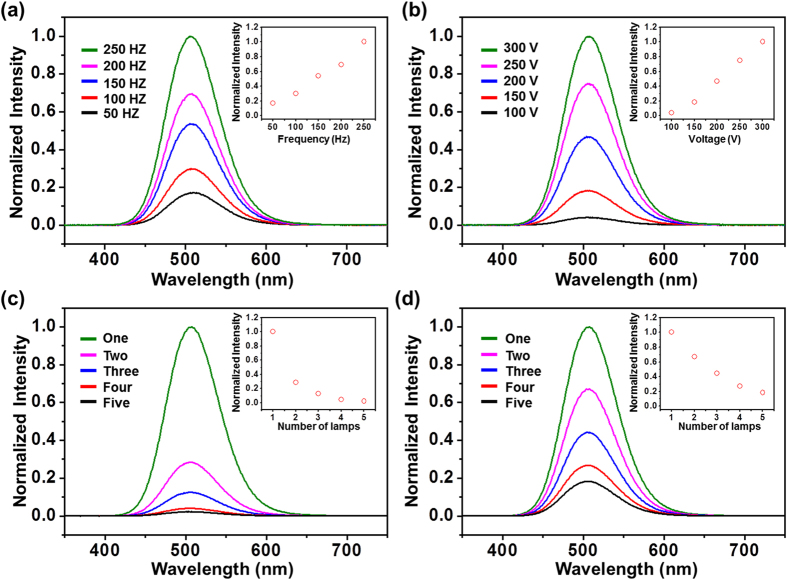
Measurement results of electroluminescence spectrum of the system when driven by the rotary TEG. (**a**) Normalized intensity of the electroluminescence spectrum at different driving frequencies. Inset: peak normalized intensity as a function of the frequency. (**b**) Normalized intensity of the electroluminescence spectrum at different driving voltages. Inset: peak normalized intensity as a function of the voltage. Normalized intensity of the electroluminescence spectrum when multiple lamps in parallel connection. (**c**) and in serial connection (**d**) are used. Insets: peak normalized intensity as a function of the number of lamps.

Factors that may influence the luminescence intensity of the self-powered system were investigated by using the rotary TEG. First, the current frequency determines the luminescence intensity to a large extent. As shown in Fig. 3a, higher frequency leads to higher output of the luminescence, which is attributed to the faster-changing electric field that can accelerate electrons in the phosphor to a larger extent^23^. Since the current frequency is controlled by the rotation rate of the TEG, the input from external mechanical energy then plays a critical role in affecting the luminescence intensity. Second, the open-circuit voltage of the TEG is another important governing factor in that higher voltage means more induced charges pumped onto the ACTFEL lamp and thus higher electric filed for exciting the photon emission^24^. As the open-circuit voltage increases from 100 V to 300 V, the luminescence intensity experiences a 25-fold enhancement, as demonstrated in Fig. 3b. Third, when multiple ACTFEL lamps (1 cm by 1 cm) are used as a load simultaneously, the way they are connected can also considerable influence the luminescence intensity. If parallel connection is employed, the intensity drops exponentially as more lamps are added. Compared to the case of a single lamp, only 2.2% of the luminescence intensity can be obtained when five lamps of the same size are connected in parallel (Fig. 3c). On the contrary, serial connection is much more favorable for light output of the self-powered system. As shown in Fig. 3d, as much as 18.3% of the luminescence intensity can be still preserved even when five ACTFEL lamps are used.

**Figure 4:**
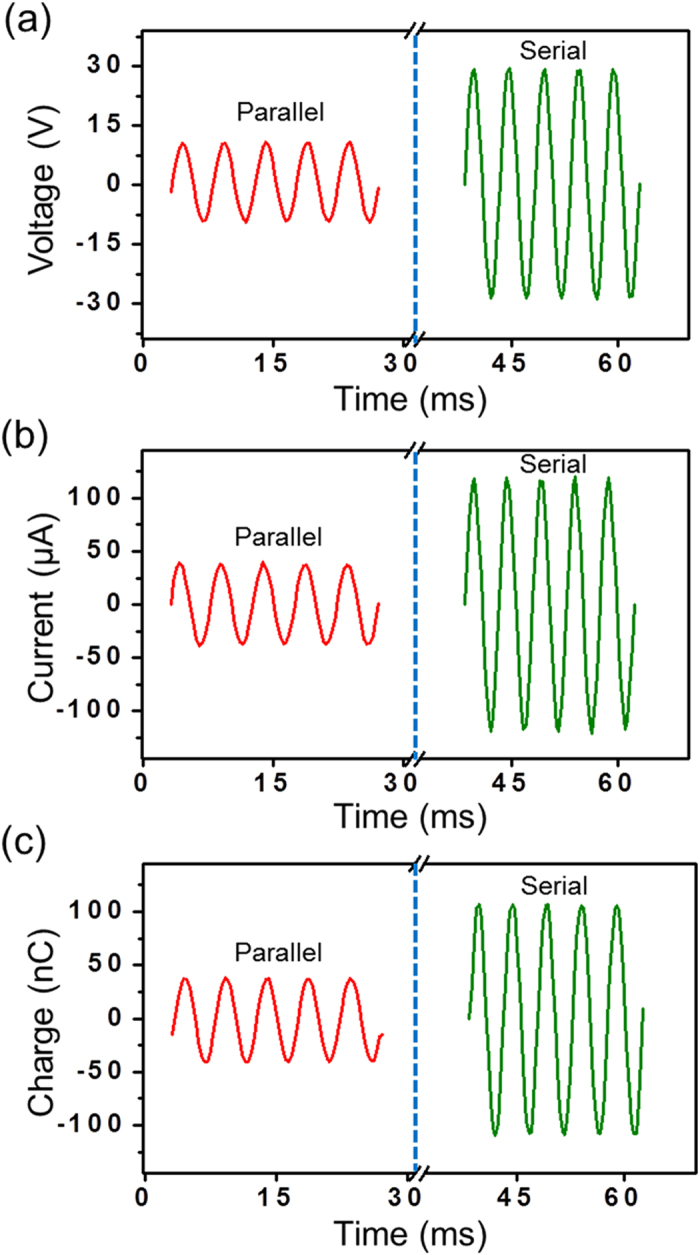
Electrical measurement results on a single ACTFEL lamp when an array of three lamps is used with different connection methods. (**a**) Voltage applied onto a lamp that is in parallel (left column) and in serial (right column) connection with others. Current (**b**) and induced charges (**c**) that flow through a lamp when it is in parallel (left column) and in serial (right column) connection with others. Note: lamps are driven by the rotary TEG at a rotation rate of 500 r/min.

**Figure 5:**
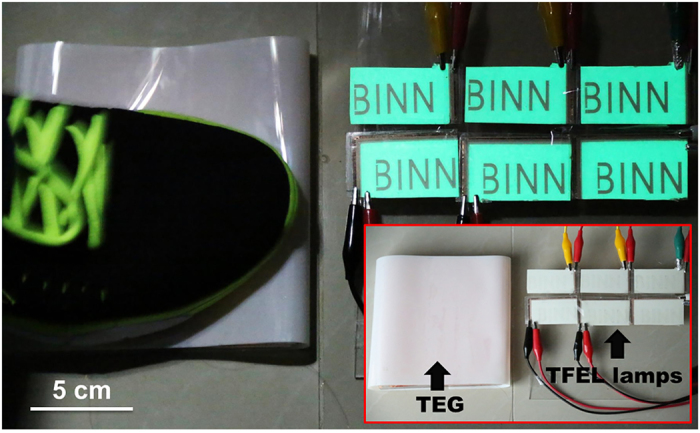
Demonstration of the area-scalable electroluminescent system when triggered by footsteps. All of the ACTFEL lamps are in serial connection. Inset: configuration of the self-powered system.

The above contradiction is attributed to different electrical characteristics of the connection methods. To illustrate this point, electrical measurement was performed on three ACTFEL lamps of the same size (3 cm by 3 cm) that were driven by a rotary TEG. The voltage applied onto each lamp for the serial connection is approximately three times of that for the parallel connection (Fig. 4a). The same result also applies to the cases of current (Fig. 4b) and induced charges (Fig. 4c). The detailed experimental values of the electrical measurement are tabulated in Supporting Table S1. Therefore, the serial connection provides a viable route in obtaining an area-scalable self-powered electroluminescent system. As demonstrated in Fig. 5, a contact TEG of 12 cm by 15 cm was directly connected to six ACTFEL lamps (3 cm by 5 cm) that were in serial connection. When triggered by footsteps, all of the lamps were excited simultaneously, which was clearly visible even in ambient light (Fig. 5 and Supporting Movie S2). The overall luminescent area reaches approximately 90 cm^2^. The demonstration reveals potentially wide applications of the self-powered system in areas such as illumination, display, indication, monitoring and surveillance.

In summary, we present a self-powered electroluminescent system without an electrical interface. The capacitor-structured ACTFEL lamp can take full advantage of the alternating high voltage from the TEG. Induced charges pumped by the TEG onto the ACTFEL can produce a fast-changing electric field across the phosphor layer to excite luminescence. Multiple types of TEGs are demonstrated as an effective power source for the system, showing the capability of the system in harnessing diverse mechanical sources. Rational serial connection of the ACTFELs enables area-scalable operation of the system, which promises wide applications.

## Methods

### Fabrication process of a contact-mode TEG

A contact-mode TEG is composed of two parts connected by elastic braces. Each part has a substrate, a back electrode and an electrification layer. (1) cut two pieces of acrylic glass with dimensions of 3 cm by 3 cm by 0.2 cm using laser cutter to serve as substrates; (2) prepare Teflon and nylon films with a uniform thickness of 30 um into 3 cm by 3 cm as a pair of electrification layers; (3) deposit a copper layer of 100 nm in thickness on one side of Teflon and nylon films by magnetron sputtering as back electrodes; (4) adhere Teflon and nylon films separately onto the substrates with the uncoated side upward; (5) connect a lead wire to each of the electrodes for measurement; (6) attach two pieces of polyimide films with dimensions of 3 cm by 3 cm by 0.125 cm to the substrates at opposite edges as elastic braces.

### Fabrication process of a rotary TEG

A rotary TEG is mainly composed a stator and a rotator. Stator: (1) manufacture a copper coated epoxy glass disc with a radius of 75 mm into two complementary patterns as separated electrodes; (2) two lead wires were separately connected to the independent electrodes for measurement; (3) adhere a layer of polytetrafluoroethylene (PTFE) film with a thickness of 30 um as an electrification layer. Rotator: (1) prepare a copper coated epoxy glass disc with a radius of 75 mm into 90 radially-arrayed sectors with designed pattern.

### Fabrication process of the ACTFEL lamps

(1) apply scotch tape along the two opposite edges of a square-shaped glass deposited with an ITO layer; (2) apply the phosphor paste, dielectric paste and silver paste (provided by vendors) in sequence using a spatula; thin them by scraping a single slide across the layer; (3) dry and cure each layer before application of the next. Each layer is dried in an oven at 130 °C for 15 minutes; (4) small pieces of copper tape are attached to the ITO layer and the silver layer separately as two electrodes.

## Additional Information

**How to cite this article**: Wei, X. Y. *et al.* Interface-Free Area-Scalable Self-Powered Electroluminescent System Driven by Triboelectric Generator. *Sci. Rep.* **5**, 13658; doi: 10.1038/srep13658 (2015).

## Supplementary Material

Supplementary Information

Supplementary Information

Supplementary Information
