# Supplementary material for: Interface-Free Area-Scalable Self-Powered Electroluminescent System Driven by Triboelectric Generator
Source: Sci Rep. 2015 Sep 4;5:13658. doi: 10.1038/srep13658 (PMC4559893; doi:10.1038/srep13658)
Supplement: Supplementary Information [file srep13658-s1.pdf]

Supporting Information

Interface-Free Area-Scalable Self-Powered Electroluminescent System Driven by Triboelectric Generator

Xiao Yan Wei[1], *Shuang Yang Kuang*[1], *Hua Yang Li*[1], *Caofeng Pan*[1], *Guang Zhu*[1]*, *Zhong Lin Wang*[1,2]

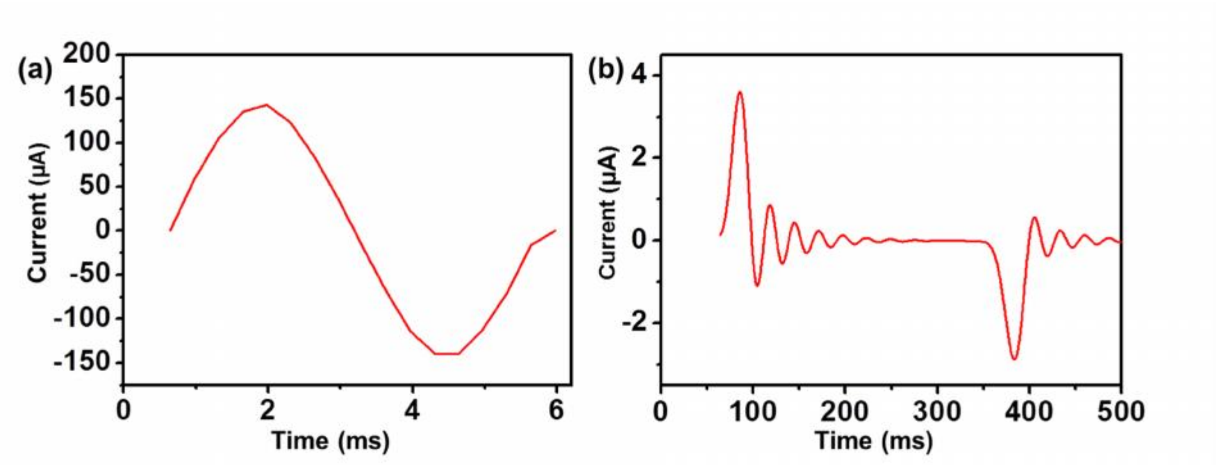

Figure S1. The Current frequency difference provided by two types of TEGs. (a) The time span of a current peak produced by the rotary TEG. (b) The time span of a current peak produced by the contact TEG.

Table S1. The detailed experimental values of the electrical measurement.

	Parallel connection	Series connection
Voltage（V）	20.8	60
Current (μA)	42	120
Charge (nC)	76	210

Movie S1. Self-Powered Electroluminescence Driven by Rotary TEG (Permission is granted from the logo copyright holder).

Movie S2. Area-Scalable Electroluminescence Triggered by Foot Steps.
